# Proestrus Differentially Regulates Expression of Ion Channel and Calcium Homeostasis Genes in GnRH Neurons of Mice

**DOI:** 10.3389/fnmol.2019.00137

**Published:** 2019-05-31

**Authors:** Csaba Vastagh, Norbert Solymosi, Imre Farkas, Zsolt Liposits

**Affiliations:** ^1^Laboratory of Endocrine Neurobiology, Institute of Experimental Medicine, Hungarian Academy of Sciences, Budapest, Hungary; ^2^Centre for Bioinformatics, University of Veterinary Medicine, Budapest, Hungary; ^3^Department of Neuroscience, Faculty of Information Technology and Bionics, Pázmány Péter Catholic University, Budapest, Hungary

**Keywords:** GnRH, ion channels, gene expression, mouse, neurons, proestrus, transcriptome

## Abstract

In proestrus, the changing gonadal hormone milieu alters the physiological properties of GnRH neurons and contributes to the development of the GnRH surge. We hypothesized that proestrus also influences the expression of different ion channel genes in mouse GnRH neurons. Therefore, we performed gene expression profiling of GnRH neurons collected from intact, proestrous and metestrous GnRH-GFP transgenic mice, respectively. Proestrus changed the expression of 37 ion channel and 8 calcium homeostasis-regulating genes. Voltage-gated sodium channels responded with upregulation of three alpha subunits (*Scn2a1*, *Scn3a*, and *Scn9a*). Within the voltage-gated potassium channel class, *Kcna1*, *Kcnd3*, *Kcnh3*, and *Kcnq2* were upregulated, while others (*Kcna4*, *Kcnc3*, *Kcnd2*, and *Kcng1*) underwent downregulation. Proestrus also had impact on inwardly rectifying potassium channel subunits manifested in enhanced expression of *Kcnj9* and *Kcnj10* genes, whereas *Kcnj1*, *Kcnj11*, and *Kcnj12* subunit genes were downregulated. The two-pore domain potassium channels also showed differential expression with upregulation of *Kcnk1* and reduced expression of three subunit genes (*Kcnk7*, *Kcnk12*, and *Kcnk16*). Changes in expression of chloride channels involved both the voltage-gated (*Clcn3* and *Clcn6*) and the intracellular (*Clic1*) subtypes. Regarding the pore-forming alpha-1 subunits of voltage-gated calcium channels, two (*Cacna1b* and *Cacna1h*) were upregulated, while *Cacna1g* showed downregulation. The ancillary subunits were also differentially regulated (*Cacna2d1*, *Cacna2d2*, *Cacnb1*, *Cacnb3*, *Cacnb4*, *Cacng5*, *Cacng6*, and *Cacng8*). In addition, ryanodine receptor 1 (*Ryr1*) gene was downregulated, while a transient receptor potential cation channel (*Trpm3*) gene showed enhanced expression. Genes encoding proteins regulating the intracellular calcium homeostasis were also influenced (*Calb1*, *Hpca*, *Hpcal1*, *Hpcal4*, *Cabp7*, *Cab 39l*, and *Cib2*). The differential expression of genes coding for ion channel proteins in GnRH neurons at late proestrus indicates that the altering hormone milieu contributes to remodeling of different kinds of ion channels of GnRH neurons, which might be a prerequisite of enhanced cellular activity of GnRH neurons and the subsequent surge release of the neurohormone.

## Introduction

The hypothalamo-pituitary-gonadal (HPG) neuroendocrine axis has a pivotal role in regulation of reproduction (Knobil, 1988) and maintenance of trophic gonadal hormone supply for different hormone sensitive cellular constituents of the body (Heldring et al., 2007). At the hypothalamic level of regulation, gonadotropin-releasing hormone (GnRH)-synthesizing neurons are in charge of initiating the reproductive hormone cascade events (Carmel et al., 1976; Merchenthaler et al., 1980). They also receive information about the functional performance of the controlled units via hormone feedback mechanisms (Sarkar and Fink, 1980; Herbison, 1998). Estrogen hormones are powerful regulators of the HPG axis acting via nuclear and membrane receptors. In female rodents, estradiol (E2) exerts both negative and positive feedback effects on GnRH neurons and their neuronal afferents including the kisspeptin system (Sarkar and Fink, 1980; Herbison, 1998; Moenter et al., 2003b; Radovick et al., 2012; Adams et al., 2018). Estrogen receptor beta (ERβ) (Hrabovszky et al., 2000, 2001) and GPR 30 (Noel et al., 2009), both produced in GnRH neurons, can sense the gonadal cycle-dependent changes of circulating E2 and adjust accordingly the cellular activity of GnRH neurons (Wang et al., 1995; Gore and Roberts, 1997; Finn et al., 1998; Christian et al., 2005; Farkas et al., 2013). The changing molecular and cellular events determine, among others, the production rate, axonal transport and release pattern of GnRH (Wang et al., 1995; Gore and Roberts, 1997; Finn et al., 1998; Christian et al., 2005; Farkas et al., 2013). The pulsatile secretion of GnRH and its pre-ovulatory surge release are characteristic features of the system (Moenter et al., 2003a). Disturbances in pulsatile and surge release mechanisms result in anovulatory syndromes and infertility (Henderson et al., 1976; Schally et al., 1976).

In proestrus, the changing gonadal hormone milieu contributes to the development of the pre-ovulatory surge release of GnRH (Clarke and Cummins, 1985; Levine, 1997; Christian and Moenter, 2010; Radovick et al., 2012). It has recently been shown that GnRH neurons exhibit burst-type firing pattern in late proestrus (Farkas et al., 2013; Silveira et al., 2017) and E2 modulates the oscillations and increases the firing (Chu et al., 2012). Proestrus also causes the remodeling of different classical neurotransmitter and neuropeptide receptors of both ionotropic and metabotropic types in GnRH neurons (Vastagh et al., 2016). These events reflect that several neuronal regulators of the GnRH system are also subject of the positive E2 feedback regulation (Gore, 2010; Wang et al., 2016) and the effects of estrogen are relayed to GnRH neurons via altered patterns of neurotransmission. The study has been focused solely on GnRH neurons that do not express estrogen receptor (ER) alpha in mice. Regarding the positive estrogen feedback-targeted kisspeptin neurons that express both isotypes of ER, we have recently shown that proestrus heavily upregulates Kiss1 in the medial preoptic area of mice (Vastagh and Liposits, 2017).

The electrophysiological properties and activity of GnRH neurons highly depend on the actual functional state of their different ion channels. The contribution of different sodium, potassium, calcium and chloride channel activities to the resting state and activation of GnRH neurons has comprehensively been studied (Van Goor et al., 1999; Costantin and Charles, 2001; Bosch et al., 2002, 2013; DeFazio and Moenter, 2002; Kelly et al., 2003; Nunemaker et al., 2003; Toba et al., 2005; Chu and Moenter, 2006; Kato et al., 2006, 2009; Farkas et al., 2007; Spergel, 2007; Zhang et al., 2007, 2009, 2013; Hiraizumi et al., 2008; Liu and Herbison, 2008; Xu et al., 2008; Krsmanovic et al., 2010; Lee et al., 2010; Moenter, 2010; Ronnekleiv et al., 2010, 2012, 2015; Arroyo et al., 2011; Pielecka-Fortuna et al., 2011; Iremonger and Herbison, 2012; Zhang and Spergel, 2012; Norberg et al., 2013). The expression of certain ion channel genes and particular ion currents generated in GnRH neurons have been found estrogen sensitive (DeFazio and Moenter, 2002; Moenter et al., 2003a; Zhang et al., 2007, 2009; Ronnekleiv et al., 2010, 2015; Wang et al., 2010; Pielecka-Fortuna et al., 2011; Bosch et al., 2013). The majority of the studies has been carried out in ovariectomized, E2-replaced animals that only partially mimic the physiological events occurring during the natural ovarian cycle.

Therefore, the present study was aimed at deciphering the biological effects of proestrus exerted upon expression of ion channels genes of GnRH neurons collected from intact, regularly cycling GnRH-GFP mice shortly before the onset of GnRH surge. The explored differential expression of various ion channel genes gives a better insight into the specific types of ion channels responding to proestrus-related hormonal events and also their reasonable contribution to development of the preovulatory GnRH surge.

## Materials and Methods

### Ethics Statement

This study was carried out in accordance with legal requirements of the European Community (Directive 2010/63/EU). The protocol was approved by the Animal Welfare Committee of the Institute of Experimental Medicine Hungarian Academy of Sciences, Budapest, Hungary (Permission Number: A5769-01). The animal experimentations were conducted in accordance with accepted standards of humane animal care and all efforts were made to minimize suffering.

### Animals

Adult, gonadally intact female mice were used from local colonies bred at the Medical Gene Technology Unit of the Institute of Experimental Medicine (IEM). They were housed in light (12:12 light-dark cycle, lights on at 06:00 h) – and temperature (22 ± 2°C) controlled environment, with free access to standard food and tap water. GnRH-green-fluorescent protein (GnRH-GFP) transgenic mice (Suter et al., 2000) bred on a C57BL/6J genetic background were used. In this animal model, a GnRH promoter segment drives selective GFP expression in the majority of GnRH neurons. The estrous cycle was monitored daily between 9 and 10 am by microscopic evaluation of vaginal cytology (Byers et al., 2012). Proestrous (*n* = 6) and metestrous (*n* = 6) female mice with at least two consecutive, regular estrous cycles were used. In order to avoid the possible circadian effect, animals were sacrificed at the same period of the day, between 16:00 and 18:00 h. Those animals were considered to be in the proestrous stage that fulfilled the following criteria: (1) vaginal smear staining with predominance of nucleated epithelial cells (Byers et al., 2012); (2) LH serum concentrations >5 ng/ml (15.11 ± 3.4 ng/ml); (3) uterus wet weights > 0.15 g (0.19 ± 0.01 g). Accordingly, the following criteria were applied for the metestrous cycle phase: (1) vaginal smears consisting of the three cell types: leukocytes, cornified, and nucleated epithelial cells (Byers et al., 2012); (2) serum LH levels < 0.5 ng/ml (0.35 ± 0.02 ng/ml); (3) uterus wet weights < 0.1 g (0.08 ± 0.01 g).

### Serum LH Measurements

Blood samples were collected from the heart of deeply anesthetized mice immediately before the brain fixation step. The samples were chilled on ice, centrifuged at 1,300 *g* for 3 min at 4°C. Plasma was aspirated then frozen and stored at −80°C until further use. Serum LH concentrations were measured with a rodent LH ELISA kit #ERK R7010 from Endocrine Technologies Inc. (Newark, CA, United States) according to manufacturers’ instructions.

### Laser Capture Microdissection, RNA Isolation and Whole Transcriptome Amplification (WTA)

Brain fixation, preparation of sections for the subsequent laser capture microdissection (LCM) and microarray profiling were performed as reported elsewhere (Khodosevich et al., 2007; Vastagh et al., 2015). Briefly, metestrous (*n* = 6) and proestrous female (*n* = 6) mice were deeply anesthetized with ketamine/xylazine (100 and 10 mg/kg body weight, respectively) and perfused transcardially with 80 ml 0.5% paraformaldehyde followed by 20% sucrose. For microdissection, 7 μm thick coronal brain sections were cut using a CM3050S cryostat (Leica, Wetzlar, Germany). Sections were mounted on PEN-membrane slides (Zeiss, Jena, Germany), processed further for laser microdissection. Uniform and representative sampling of the entire GnRH neuronal population was performed using LCM performed on a PALM Microbeam system (Carl Zeiss Microimaging Gmbh, Jena, Germany) which was equipped with an epifluorescent setup. 250 GFP-positive neurons were dissected and pooled from 80 to 100 consecutive sections of each brain.

GnRH cell samples collected with LCM were incubated in 200 μl lysis buffer at 56°C for 3 h. RNA was isolated from the lysate by proteinase K/acid phenol method. RNA was purified using RNeasy MinElute Cleanup kit (Qiagen, Hilden, Germany). Total RNA was eluted with 14 μl of ribonuclease-free water. The quality of RNA was measured with Bioanalyzer. The integrity of the isolated RNA (RIN values: 6.8–7.2) was proven sufficient for the subsequent amplification steps.

Library preparation and amplification were performed according to the manufacturer’s (Sigma-Aldrich) instructions for the WTA2 kit. When the SYBR Green signal reached a plateau, the reaction was stopped. The yielded cDNA (fragment length: 100–1,000 bp, amount: 7–8 microgram) met the criteria of Pico profiling of low cell numbers (Gonzalez-Roca et al., 2010). The amplified double-stranded cDNA was purified and quantified on a Nanodrop ND-1000 spectrophotometer (Thermo Fisher Scientific, Waltham, MA, United States).

### Mouse Genome 430 PM Arrays

Eight μg cDNA was fragmented by DNase I and biotinylated by terminal transferase obtained from the GeneChip Mapping 250K Nsp Assay Kit (Affymetrix Inc., Santa Clara, CA, United States). Hybridization, washing, staining and scanning of Affymetrix Mouse Genome 430 PM Strip arrays were performed following the manufacturer’s recommendations. The Mouse Genome 430 PM Strip array allows the analysis of 34,325 well-annotated genes using 45,123 distinct probe sets. Scanned images (DAT files) were transformed into intensities (CEL files) using the AGCC software (Affymetrix). RMA analysis was performed by means of the statistical analysis software Partek Genomics Suite (Partek Inc., St. Louis, MO, United States) to obtain probe set level expression estimates.

### Bioinformatics, Data Analysis

All statistical and data mining works were performed in R-environment (R Core Team, 2018) with Bioconductor packages (Huber et al., 2015). Quality assessment of microarrays (*n* = 12) was performed using affyQCReport. Raw microarray data were pre-processed for analysis using RMA (Robust Multi-Array Average) (Irizarry et al., 2003). Fold change (FC) estimation and difference analysis of gene expression were based on linear models combined with Bayesian methods. FC was calculated from normalized and log_2_ transformed gene expression microarray data for each probe sets. The obtained *p*-values were adjusted by the FDR-based method. The following cut-off criteria were applied on the differentially expressed genes: fold change >1.5; and adjusted *p*-value (*p*_adj_) <0.05.

The differentially regulated genes were displayed in heat map. KEGG pathway analysis^1^ was used to reveal the main gene ontology (GO) pathways associated with molecular functions linked to the differentially expressed genes (DEGs). The putative interactions among proteins encoded by DEGs were analyzed by the web-based STRING v10.5 program^2^ (Szklarczyk et al., 2015).

### Validation of Microarray Data With Quantitative Real-Time PCR Studies

For quantitative real-time PCR (qPCR) investigations of LCM-derived GnRH samples (proestrous females *n* = 6, metestrous *n* = 5) RNA isolation and WTA were performed as described in the previous section. Amplified and column-purified cDNA was used as template for qPCR. Whole transcriptome-amplified cDNA from LCM samples were diluted in 0.1× TE buffer for qPCR investigation. Inventoried TaqMan assays were used to confirm microarray results by qPCR. Each assay consisted of a FAM dye-labeled TaqMan MGB probe and two PCR primers. Thermal cycling conditions of the qPCR were as follows: 2 min at 50°C and 10 min at 95° C, followed by 40 cycles of 15 s at 95°C and 1 min at 60°C using ViiA 7 real-time PCR platform (Life Technologies). Relative gene expression (RQ) was calculated by the 2^–ΔΔCt^ method (Livak and Schmittgen, 2001) using GAPDH gene as a reference.

The log2 transformed RQ (qPCR), FC (microarray) values were scatter-plotted (Statistica ver. 13; Tibco Software, Palo Alto, CA, United States), and linear regression analysis was performed using R software (R Core Team, 2018).

## Results

In this work, we examined the influence of proestrus on the expression of genes encoding various ion channel proteins and regulators of intracellular calcium homeostasis in GnRH neurons dissected from intact, metestrous and proestrous GnRH-GFP transgenic mice brains, respectively.

**FIGURE 1:**
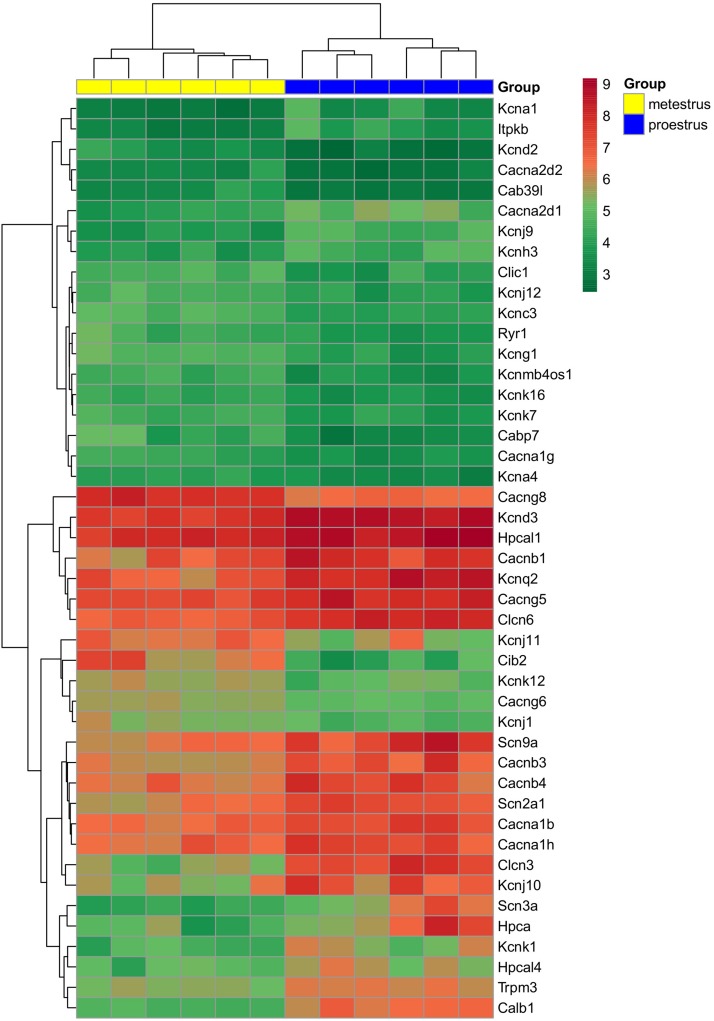
Heat map of genes regulated differentially in GnRH neurons of proestrous versus metestrous mice. Expression levels of genes coding ion channels and regulators of calcium homeostasis. The rows represent differentially expressed probe sets with corresponding gene symbols on the right. The expression level of each probe is color coded. For decoding, see the color key. The individual samples are shown as columns. The six metestrous and six proestrous samples are coded in yellow and blue, respectively.

**FIGURE 2:**
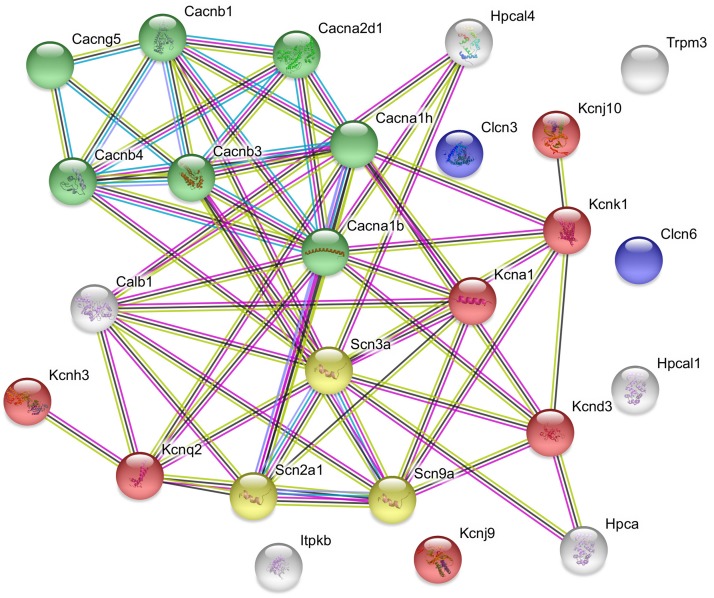
Predicted interactions among proteins encoded by upregulated ion channel/transport genes in GnRH neurons of proestrous mice. The gene network was constructed by using the STRING 10.5 Known and Predicted Protein–Protein Interactions program (http://string-db.org/). Evidence view, medium confidence. The majority of the proteins (19) form functional clusters belonging to voltage-gated sodium (yellow), potassium (red), and calcium (green) channels. Six proteins represent the non-networking group (Clcn3, Clcn6, Trpm3, Hpcal1, Kcnj9, and Itpkb). Color code of highlighted proteins: red: voltage-gated potassium channel, green: voltage-gated calcium channel, yellow: voltage gated sodium channel, blue: voltage-gated chloride channel. Color code for lines in evidence view: red line – presence of fusion evidence, green line – neighborhood evidence, blue line – co-occurrence evidence, purple line – experimental evidence, light blue line – database evidence, black line co-expression.

**FIGURE 3:**
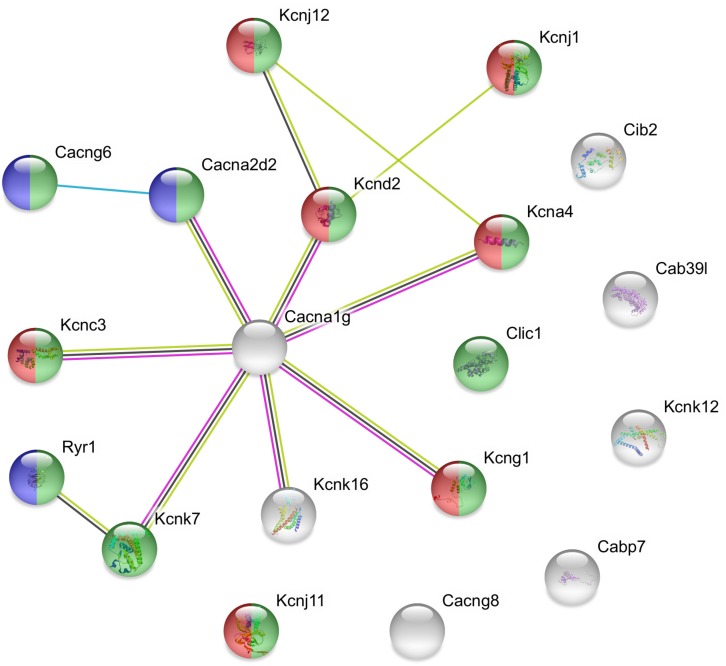
Predicted interactions among proteins encoded by downregulated ion channel/transport genes in GnRH neurons of proestrous mice. The gene network was constructed by using the STRING 10.5 Known and Predicted Protein–Protein Interactions program (http://string-db.org/). Evidence view, medium confidence. It contains 13 networking proteins representing predominantly calcium channel (blue), voltage-gated channel (green), and voltage-gated potassium channel activities (red). The non-networking proteins numbered 6 (Cib2, Cab39l, Kcnk12, Cabp7, Cacng8, and Kcnj11). Color code of highlighted genes: green: voltage-gated channel activity, red: voltage-gated potassium channel activity, blue: calcium channel activity. Color code for lines in evidence view: red line –presence of fusion evidence, green line – neighborhood evidence, blue line – co-occurrence evidence, light blue line – database evidence, black line co-expression.

The used Mouse Genome 430 PM Strip Array allowed the analysis of 20563 genes. The total number of differentially regulated genes was 5791. Kegg pathway analysis and hand-picking were used for forming the “ion channel proteins and regulators of calcium homeostasis” group with 85 members. At the used cut-off values [fold change >1.5; and adjusted *p*-value (*p*_adj_) <0.05], the final group numbered 45 genes. Twenty-five of them were upregulated (Table 1). The differential expression of individual genes was displayed in heat map (Figure 1). The top gene ontology (GO) “molecular function” pathways linked to the differentially expressed genes are summarized in Table 2. The predicted interactions among proteins encoded by the DEGs in GnRH neurons of late proestrous mice are depicted in Figures 2, 3, as up- and downregulated clusters, respectively.

**TABLE 1:** Differentially expressed genes encoding ion channels and regulators of calcium homeostasis in GnRH neurons of proestrous mice.

**Probe ID**	**Gene symbol**	**Gene synonym**	**Description**	**FC**	**adj *p*-val**
**Sodium channel**					
1439204_at	Scn3a	Nav1.3	Sodium channel, voltage-gated, type III, alpha	**3.33**	6.23E-03
1442333_a_at	Scn9a	Nav1.7	Sodium channel, voltage-gated, type IX, alpha	**2.46**	1.10E-02
1427280_at	Scn2a1	Nav1.2	Sodium channel, voltage-gated, type II, alpha 1	**1.98**	3.71E-03
**Potassium channel**					
1440258_at	Kcnq2	Kv7.2	Potassium voltage-gated channel, subfamily Q, member 2	**2.68**	2.56E-03
1419601_at	Kcnj10	Kir4.1	Potassium inwardly rectifying channel, subfamily J, member 10	**2.59**	1.46E-02
1448690_at	Kcnk1	K2p1.1	Potassium channel, subfamily K, member 1	**2.06**	2.20E-02
1426070_a_at	Kcnd3	Kv4.3	Potassium voltage-gated channel, Shal-related family, member 3	**2.02**	6.94E-04
1450712_at	Kcnj9	Kir3.3	Potassium inwardly rectifying channel, subfamily J, member 9	**1.84**	2.87E-03
1455785_at	Kcna1	Kv1.1	Potassium voltage-gated channel, shaker-related subfamily, member 1	**1.80**	4.80E-02
1459107_at	Kcnh3	Kv3.3	Potassium voltage-gated channel, subfamily H (eag-related), member 3	**1.59**	3.23E-02
1429913_at	Kcnk16	K2p16.1	Potassium channel, subfamily K, member 16	0.66	5.95E-03
1425437_a_at	Kcnk7	K2p7.1	Potassium channel, subfamily K, member 7	0.66	1.19E-02
1422871_at	Kcnj12	Kir.2.2	Potassium inwardly rectifying channel, subfamily J, member 12	0.65	1.46E-02
1422255_at	Kcna4	Kv1.4	Potassium voltage-gated channel, shaker-related subfamily, member 4	0.65	5.98E-03
1421981_at	Kcnc3	Kv3.3	Potassium voltage gated channel, Shaw-related subfamily, member 3	0.61	1.25E-03
1418614_at	Kcnj1	Kir1.1	Potassium inwardly rectifying channel, subfamily J, member 1	0.60	7.91E-03
1441280_at	Kcnk12	K2p12.1	Potassium channel, subfamily K, member 12	0.59	1.14E-02
1453449_at	Kcnmb4os1		Potassium large conductance calcium-activated channel, subfamily M, beta member 4, opposite strand 1	0.56	3.72E-03
1459656_at	Kcng1	Kv6.1	Potassium voltage-gated channel, subfamily G, member 1	0.55	2.01E-03
1447764_at	Kcnd2	Kv4.2	Potassium voltage-gated channel, Shal-related family, member 2	0.51	3.72E-03
1455417_at	Kcnj11	Kir.6.2	Potassium inwardly rectifying channel, subfamily J, member 11	0.50	2.86E-02
**Chloride channel**					
1438366_x_at	Clcn3		Chloride channel 3	**4.53**	2.42E-04
1422314_at	Clcn6		Chloride channel 6	**2.27**	2.81E-04
1416656_at	Clic1		Chloride intracellular channel 1	0.61	1.79E-02
**Calcium channel**					
1426108_s_at	Cacnb1		Calcium channel, voltage-dependent, beta 1 subunit	**2.11**	4.13E-02
1448656_at	Cacnb3		Calcium channel, voltage-dependent, beta 3 subunit	**2.08**	1.04E-02
1433643_at	Cacna2d1		Calcium channel, voltage-dependent, alpha2/delta subunit 1	**1.99**	7.15E-03
1452089_at	Cacnb4		Calcium channel, voltage-dependent, beta 4 subunit	**1.96**	2.76E-02
1441966_at	Trpm3		Transient receptor potential cation channel, subfamily M, member 3	**1.72**	7.28E-04
1426330_at	Cacng5		Calcium channel, voltage-dependent, gamma subunit 5	**1.70**	6.23E-03
1425812_a_at	Cacna1b	Cav2,2	Calcium channel, voltage-dependent, N type, alpha 1B subunit	**1.68**	9.04E-03
1422710_a_at	Cacna1h	Cav3.2	Calcium channel, voltage-dependent, T type, alpha 1H subunit	**1.65**	3.86E-02
1427306_at	Ryr1		Ryanodine receptor 1, skeletal muscle	0.61	2.12E-02
1425730_at	Cacng6		Calcium channel, voltage-dependent, gamma subunit 6	0.65	6.92E-04
1426185_at	Cacna2d2		Calcium channel, voltage-dependent, alpha 2/delta subunit 2	0.64	1.74E-02
1423365_at	Cacna1g	Cav3.1	Calcium channel, voltage-dependent, T type, alpha 1G subunit	0.63	6.43E-03
1451864_at	Cacng8		Calcium channel, voltage-dependent, gamma subunit 8	0.39	5.34E-05
**Calcium homeostasis**					
1417504_at	Calb1		Calbindin 1	**3.49**	2.65E-05
1450930_at	Hpca		Hippocalcin	**3.41**	2.96E-02
1435272_at	Itpkb		Inositol 1,4,5-trisphosphate 3-kinase B	**1.88**	1.79E-02
1448812_at	Hpcal1		Hippocalcin-like 1	**1.76**	9.06E-03
1433987_at	Hpcal4		Hippocalcin-like 4	**1.75**	3.25E-02
1446539_at	Cab39l		Calcium binding protein 39-like	0.62	1.12E-02
1425963_at	Cabp7		Calcium binding protein 7	0.45	8.49E-03
1447770_at	Cib2		Calcium and integrin binding family member 2	0.22	2.32E-03

**List of differentially expressed genes (DEGs). FC values indicate the changes of gene expression in proestrous versus metestrous GnRH neurons. Gene symbols and FC values of upregulated genes are in bold. FC, fold change; adj.P.Val, adjusted P-value*.*

**TABLE 2:** Gene ontology.

**Molecular function (GO)**		
**Pathway ID**	**Pathway description**	**Count in gene set**
GO:0005244	Voltage-gated ion channel activity	31
GO:0005261	Cation channel activity	28
GO:0022843	Voltage-gated cation channel activity	24
GO:0046873	Metal ion transmembrane transporter activity	28
GO:0022857	Transmembrane transporter activity	32
GO:0005267	Potassium channel activity	17
GO:0005249	Voltage-gated potassium channel activity	15
GO:0015077	Monovalent inorganic cation transmembrane transporter activity	18
GO:0005262	Calcium channel activity	10
GO:0005245	Voltage-gated calcium channel activity	8
GO:0005242	Inward rectifier potassium channel activity	6
GO:0008331	High voltage-gated calcium channel activity	4
GO:0015276	Ligand-gated ion channel activity	7
GO:0015272	ATP-activated inward rectifier potassium channel activity	3
GO:0005251	Delayed rectifier potassium channel activity	4
GO:0031420	Alkali metal ion binding	3
GO:0016247	Channel regulator activity	5
GO:0005250	A-type (transient outward) potassium channel activity	2
GO:0005509	Calcium ion binding	8
GO:0044325	Ion channel binding	4
GO:0005246	Calcium channel regulator activity	3
GO:0030955	Potassium ion binding	2
GO:0022841	Potassium ion leak channel activity	2
GO:0005247	Voltage-gated chloride channel activity	2
GO:0005254	Chloride channel activity	3

**List of GO molecular function pathways affected by the 45 differentially regulated ion channel genes in GnRH neurons of proestrous mice. The top 10 pathways are characterized by large count numbers in the gene set. The top 10 pathways are characterized by large count numbers in the gene set (8–32).**

### Differential Expression of Genes Encoding Voltage-Gated Sodium Channels

Analysis of microarray data revealed differentially expressed genes encoding sodium channels. Three different ion-pore forming alpha 1 subunits of the voltage-gated sodium channels (*Scn2a1*, *Scn3a*, and *Scn9a*) were affected. All of them showed upregulation (Table 1 and Figure 1).

### Influence of Proestrus on the Expression of Potassium Channel Genes

Similar to the sodium channel-coding genes, proestrus altered heavily the expression of potassium channel genes (Table 1 and Figure 1). Altogether 18 genes displayed differential expression. Within the voltage-gated potassium channel class, 8 genes were influenced. *Kcna1*, *Kcnd3*, *Kcnh3*, and *Kcnq2* were upregulated, while *Kcna4*, *Kcnc3*, *Kcnd2*, and *Kcng1* responded with downregulation. Proestrus also had impact on inwardly rectifying potassium channel subunits manifested in increased expression of *Kcnj9* and *Kcnj10* genes, whereas *Kcnj1*, *Kcnj11*, and *Kcnj12* subunit genes showed reduced expression. The two-pore domain potassium channel subfamily also displayed differential expression with upregulation of *Kcnk1* and diminished expression of 3 subunit genes (*Kcnk7*, *Kcnk12*, and *Kcnk16*). The BK group of calcium-activated potassium channel class *per se* was not affected, only the non-coding strand of the beta 4 subunit (*Kcnmb4os*) showed downregulation in proestrus.

### Proestrus Evoked-Changes in Expression of Chloride Channel Genes

Proestrus influenced the expression of chloride channel genes (Table 1 and Figure 1). Three of them demonstrated differential expression. Two voltage-gated chloride channel genes were upregulated (*Clcn3* and *Clcn6*), while the expression of the intracellular type of chloride channel gene (*Clic1*) showed an opposite trend.

### Differential Expression of Calcium Channel-Related Genes

Altogether 13 calcium channel-related genes were differentially regulated by proestrus, 8 exhibited upregulation (Table 1 and Figure 1). From the ion pore-forming alpha 1 subunits of the voltage-dependent calcium channel, two were upregulated (*Cacna1b* and *Cacna1h*), while *Cacna1g* showed downregulation, indicating that both N- and T-types of voltage dependent calcium channel are influenced by proestrus. Genes coding for the auxiliary subunits of the channel were also differentially regulated. Two genes belonging to the alpha2 delta subunit were altered, *Cacna2d1* showed upregulation, while expression of *Cacna2d2* was decreased. Three auxiliary beta subunits displayed marked upregulation (*Cacnb1*, *Cacnb3*, and *Cacnb4*). Out of the three regulated gamma subunits, *Cacng5* increased its expression, whereas *Cacng6* and *Cacng8* were downregulated. In addition, the intracellular calcium channel coding gene, ryanodine receptor 1 (*Ryr1*) was downregulated. A transient receptor potential cation channel (*Trpm3*) gene showed enhanced expression.

### Effects of Proestrus on Regulators of Intracellular Calcium Homeostasis

In this functional category, genes coding for calbindin 1 (*Calb1*), hippocalcin (Hpca), hippocalcin-like 1 (Hpcla1), hippocalcin-like 4 (*Hpcla4*) and the calcium/calmodulin-regulated enzyme, inositol 1,4,5-trisphosphate 3-kinase B gene (*Itpkb*) were all upregulated. In contrast, other calcium binding protein genes showed downregulation (*Cabp7*, *Cab39l*, and *Cib2*).

### Validation of the Microarray Data

**FIGURE 4:**
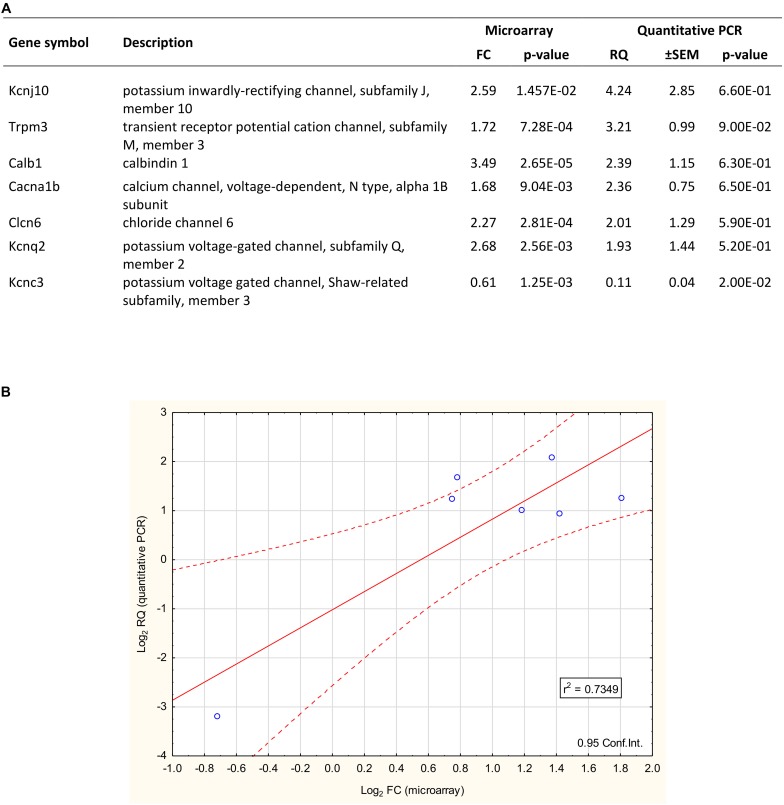
Validation of microarray data with quantitative PCR. **(A)** The qPCR confirmed the differential expression of 7 selected genes. Six showed upregulation (Kcnj10, Trpm3, Calb1, Clcn6, Cacna1b, and Kcnq2), while one gene (Kcnc3) represented the downregulated category. The fold change (FC) values of these genes – gained from the microarray dataset – are also listed. **(B)** The plot illustrates the correlation between microarray and quantitative PCR data. The coefficient of determination (*r*^2^) is 0.7349. Pearson’s product-moment correlation: (*r* = 0.8685, df = 5; *p* = 0.0137).

TaqMan real-time PCR was used for validation of microarray data (Figure 4). The expression of seven arbitrarily chosen genes was confirmed including upregulated (*Kcnj10*, *Trpm3*, *Calb1*, *Clcn6*, *Kcnq2*, and *Cacna1b*) and downregulated (*Kcnc3*) representatives of the proestrus-regulated ion channels. The coefficient of determination (*r*^2^ = 0.7349) indicates the rate of explained variation and predictable association between the log2 transformed FC (microarray) and RQ (qPCR) data. The Pearson product-moment statistics showed significant linear correlation between microarray and PCR data (Pearson’s *r* = 0.8685, df = 5, *p* = 0.0137, CI = 0.95).

## Discussion

The basic types of ion channels have been described in GnRH neurons and correlated with characteristics of different currents generated under various physiological conditions in these neurosecretory cells (Kato et al., 2009; Moenter, 2010; Bosch et al., 2013; Norberg et al., 2013). Due to the fundamental role of E2 in regulation of the HPG axis via negative and positive feedback mechanisms, the influence of this gonadal hormone on the expression and function of different ion channels has extensively been studied, mainly in ovariectomized-E2-replaced animals (DeFazio and Moenter, 2002; Nunemaker et al., 2002; Chu et al., 2009, 2010; Ronnekleiv et al., 2010; Pielecka-Fortuna et al., 2011; Bosch et al., 2013).

Choosing the preovulatory functional state of GnRH neurons in intact, cycling mice was purposeful in the study. Our intention has been to gain knowledge about changes occurring in the expression of ion channels genes in mouse GnRH neurons, as a consequence of proestrus. Accordingly, using this model, we aimed to exclude the diverse effects of ovariectomy and E2 replacement and preserve a fully functional ovarian system. OVX – in addition to ceasing the natural E2 signaling – also abolishes the supply of several other indispensable hormones, such as progesterone, activin, inhibin and anti-Müllerian hormone to the brain, including GnRH neurons and their afferents. Furthermore, the E2 replacement of OVX mice cannot totally mimic the natural fluctuation of E2. That might explain why the proestrus driven expression pattern of ion channels in GnRH neurons only partially match data published in OVX + E2 mice (Zhang et al., 2007; Bosch et al., 2013). The functional differences between the two rodent models are important and should be further clarified at both molecular and network levels of GnRH neurons. Recent electrophysiological reports are in support of the rational using intact, regularly cycling mice (Farkas et al., 2013, 2018; Adams et al., 2018).

Accordingly, in the present transcriptome study of mouse GnRH neurons, we focused on the impact of proestrus on the expression of genes coding for ion channel and calcium binding proteins. Proestrus resulted in differential expression of 45 genes. A similar robust effect of proestrus on the expression of neurotransmitter receptor genes in GnRH neurons has recently been reported (Vastagh et al., 2016).

### Proestrus Upregulates Voltage-Gated Sodium Channels

Sodium and calcium channels have been found to regulate the LH surge-generating mechanisms in the preoptic area of proestrous rats (Fukushima et al., 2003). GnRH neurons display tetrodotoxin-sensitive voltage-gated sodium channels, with a high density in the initial 150 micrometer segment of their dendrites (Iremonger and Herbison, 2012). The channels are involved in generation of the rapid depolarizing phase of the action potential and formation of slow after depolarization potential (sADP) (Chu and Moenter, 2006) that contributes to repetitive firing. Proestrus upregulated three alpha subunits [Scn2a1 (Nav1.2), Scn3a (Nav1.3) and Scn9a (Nav 1.7)] of the sodium channels without any effect on the expression of the beta subunit. These changes suggest that proestrus including the characteristic rise and peak of E2 leads to the modulation of sodium conductance in GnRH neurons. Chu and Moenter (2006) have shown that tetrodotoxin (TTX)-sensitive sodium conductance mediates the intrinsically generated sADPs which seem to contribute to repetitive firing of GnRH cells and release of the neurohormone. These events – in addition – to serving the burst firing also prolong the depolarization, change the responsiveness to network influences, alter the frequency of GnRH pulses and synchronize the network for an optimal hormone discharge (Chu and Moenter, 2006), features that also characterize the surging GnRH neurons.

### Voltage-Gated, Inwardly Rectifying and Two-Pore-Domain Potassium Channels Are Targeted by Proestrus

The different potassium conductances and their estrogen sensitivity in GnRH neurons have recently been reviewed (Norberg et al., 2013). Voltage-gated potassium currents (*I*_A_ and *I*_K_) are regulated by feedback actions of E2 (DeFazio and Moenter, 2002; Pielecka-Fortuna et al., 2011) and they are known to control the excitability and discharge activity of GnRH cells. K_ATP_ activity is also modified by E2 (Zhang et al., 2007), similar to the *S*_K_ channel (Bosch et al., 2002). GnRH neurons also exhibit BK (Hiraizumi et al., 2008) and M (Xu et al., 2008) currents, although they have been reported to lack estrogen sensitivity (Norberg et al., 2013).

### Voltage-Gated Potassium Channel

Four voltage-gated potassium channels responded to proestrus with upregulation. Kcnq2 (Kv7.2) which contributes to the formation of M-potassium channel was robustly upregulated, while the other two known constituents of M channel in GnRH neurons, Kcnq3 and Kcnq5 (Xu et al., 2008) were not altered. The M-type potassium currents are subthreshold, non-inactivating channels that diminish cell excitability (Norberg et al., 2013). They may contribute to the autoregulation of the GnRH neuronal network via a self-feedback loop (Xu et al., 2008). The enhanced Kcnq2 mRNA expression may reflect the preparation of the GnRH neuron for the forthcoming shutdown of surge-related electrophysiological events and desynchronization of the GnRH network.

The upregulated Kcnd3 (Kv4.3) shows the influence of high E2 on the expression of the Shal-related, fast inactivating A-type potassium channel. This finding is in line with a previous report showing the increased expression of Kv4.3 in GnRH neurons of diestrus-proestrus rats compared to metestrous animals (Arroyo et al., 2011).

The coding gene of a slowly inactivating, Shaker-related, K-type potassium channel, the Kcna1 (Kv1.1) also displayed upregulation, together with the Kcnh3 (Kv3.3), the slowly activating channel gene. The shaker-related subfamily of potassium channel exists in mouse GnRH neurons (Liu and Herbison, 2008). Elevated levels of intracellular calcium lead to its activation and control of the firing dynamism of neurons.

The downregulated category was comprised of mainly A type potassium channel genes, the Kcna4 (Kv1.4), Kcnc3 (Kv3.3), and Kcnd2 (Kv4.2). The modifier/silencer channel Kcng1 (Kv6.1) also showed decreased expression.

These data indicate that the high E2 level at late proestrus alters the expression of several voltage-gated potassium channel subunit genes and modifies *I*_A_, *I*_K_, and *I*_M_ types of potassium conductances in GnRH neurons that control their excitability and hormone release (DeFazio and Moenter, 2002).

### Inwardly Rectifying Potassium Channel

Proestrus exerted influence on the expression of a wide range of genes known to code inwardly rectifying potassium channels. Two members of this potassium channel subfamily displayed upregulation, Kcnj9 (Kir3.3,GIRK3) and Kcnj10 (Kir.4.1). The others were downregulated including Kcnj1 (Kir1.1), Kcnj11 (Kir6.2, K_ATP_), and Kcnj12 (Kir2.2, IRK2). Among the inwardly rectifying potassium channel proteins, Kir6.2 has comprehensively been studied in GnRH neurons (Zhang et al., 2007, 2009). Together with sulphonylurea receptor, it forms the ATP sensitive K^+^ (K_ATP_) channel. It is expressed in about 50% of GnRH neurons of both sexes. In females, the current carried by the K_ATP_ channel is controlled by E2, while the expression of Kir6.2 is not regulated by E2 (Zhang et al., 2007). Our present findings indicate that the expression of Kir. 6.2. is downregulated in proestrus.

The hyperpolarization imposed on dendrites and somata of GnRH neurons by K_ATP_ (Norberg et al., 2013) may be diminished or canceled by the decreased expression of Kir.6.2 in proestrus.

The role of G protein-gated inwardly rectifying potassium channels (GIRKs) in regulation of GnRH neurons has been demonstrated in case of galanin (Constantin and Wray, 2016) and luteinizing hormone (Hu et al., 2006) modulation.

### Two-Pore-Domain Potassium Channel

This subfamily K of potassium channel participates in coding for proteins of leak potassium channels (Enyedi and Czirjak, 2010) which contribute to the resting potential. In addition, they are also regulated by G protein-coupled receptors (Mathie, 2007). The present results clearly show that proestrus influences the expression of this potassium channel subfamily, evoking the upregulation of Kcnk1, and decreased expression of Knck7, Kcnk12, and Kcnk16. Understanding the exact role of these channels in physiology of GnRH neurons awaits further studies.

### Ca^2+^-Activated Potassium Channel

In contrast to the above described three, main potassium channel types altered by proestrus, the subunits of the large conductance calcium-activated potassium channel (BK channel) (Hiraizumi et al., 2008) were not influenced by proestrus. Only the non-coding strand of beta 4 subunit (Kcnmb4os1) showed a downregulatory response in proestrus.

### Influence of Proestrus on Chloride Channel-Coding Genes

The intracellular concentration of chloride ions in rodent GnRH neurons is maintained by several mechanisms including ligand-gated neurotransmitter receptors (GABA_A_ receptor and glycine receptor), sodium-potassium-chloride transporters and voltage-sensitive chloride channels (Han et al., 2002; Norberg et al., 2013; Taylor-Burds et al., 2015). Here, we report the upregulation of two members (Clcn3 and Clcn6) of epithelial chloride channel family (E-ClC) (Jentsch et al., 2002) by proestrus. In contrast, an intracellular chloride channel (Clic1) was downregulated in GnRH cells. Elucidation of the functional role of these chloride channel alterations requires further studies in GnRH neurons. It is noteworthy, that proestrus also intensely regulates the two neurotransmitter-gated chloride channels, the GABA_A_- and the alpha subunit of glycine receptor (Vastagh et al., 2016).

### Expression of Calcium Channels Is Regulated by Proestrus

Proestrus heavily changed the expression of voltage-gated calcium channel genes. Both ion pore- forming, voltage-sensing alpha subunits and the different auxiliary subunits (beta, alpha2-delta, and gamma) were altered. In addition, a transient receptor potential cation channel gene (Trpm3) that encodes a channel for constitutive entry of Ca^2+^ and a ryanodine receptor-coding gene (Ryr1) were also differentially regulated in proestrus.

### Effects of Proestrus on Voltage-Gated Calcium Channel Expression

Both high and low voltage-activated calcium channels were altered. Regarding the high voltage class, Cacna1b (Cav2.2) was upregulated, indicating a selective effect upon the N type channel. In the low voltage category, two T-type channels were targeted. Cacna1h (Cav3.2) was upregulated, while Cacna1g (Cav3.1) showed decreased expression. The auxiliary units of the channel also showed a marked response. Three beta subunit genes underwent upregulation (Cacnb1, Cacnb3, and Cacnb4). The alpha2 delta and gamma subunits changed their expression in both directions. Alpha2-delta 1 (Cacna2d1) and gamma subunit five (Cacng5) genes increased their expression in proestrus. In contrast, alpha2-delta 2 (Cacna2d2), gamma subunit six (Cacng6) and gamma subunit 8 (Cacng8) all showed downregulation. GnRH neurons express all four types of high voltage-activated calcium channels (L, N, P/Q, and R) and the T-type channel regulated by low voltage (Bosma, 1993; Kato et al., 2003, 2009; Nunemaker et al., 2003; Tanaka et al., 2010; Bosch et al., 2013). E2 treatment of OVX mice increased the expression of the alpha1 subunits of the T type channel and augmented the density of T calcium currents (Zhang et al., 2009). Similar to the OVX-E2 animal model, these channel events may also contribute to the burst firing of GnRH neurons in late proestrus. In another study, the low voltage-mediated calcium currents were not affected by E2, while the high voltage-activated currents, especially the L-and N-type components, were influenced by E2 with cooperation of estrogen receptor beta and GPR30 activation (Sun et al., 2010).

The skeletal muscle type ryanodine receptor (Ryr1) is widely expressed in the brain, including the hypothalamus (De Crescenzo et al., 2012). We found its expression downregulated in late proestrus. Ryr1 is coupled to L-type calcium channels and controls the voltage-induced calcium release from internal Ca^2+^ stores. Its significance in the calcium homeostasis of GnRH neurons requires further studies.

Activation of the transient receptor potential melastatin-3 (TRPM3) channel results in a rise of intracellular calcium (Thiel et al., 2017). Proestrus caused a significant upregulation of its coding gene (Trpm3). The participation of TRPM3 channel in the modulation of the intracellular calcium concentration and its putative contribution to development of calcium transients and concurrent burst firing of GnRH neurons (Constantin et al., 2012) await further studies.

### Other Regulators of Calcium Homeostasis

Proestrus differentially regulated several genes known to encode different calcium binding proteins. In the upregulated category, the coding gene of calbindin D28 (Calb1), and three gene members of the neuronal calcium sensor family coding for hippocalcin (Hipca), hippocalcin-like 1 (Hpcal1) and hippocalcin-like 4 (Hpcal4) were exposed. The inositol 1,4,5-trisphosphate 3-kinase B coding gene (Itpkb) was also upregulated. This enzyme produces the inositol 1,3,4,5-tetrakisphosphate (IP4), which regulates store-operated calcium channels (Miller et al., 2007). Other calcium binding protein-coding genes were downregulated (Cib2, Cabp7, and Cab39l). These data support the view that E2 can influence the calcium buffering capacity of GnRH neurons by altering the expression of several calcium-binding proteins.

### Predicted Interactions Among Proteins Encoded by Ion Channel and Ca-Homeostasis- Regulating Genes in GnRH Neurons of Proestrous Mouse

The STRING analysis of proestrus-altered genes/proteins revealed a coherent, upregulated cluster of voltage-gated sodium channels that is essential to depolarization and enhanced activity of GnRH neurons seen before the onset of GnRH surge. A similar strength of interaction and upregulated state characterized the subunits of voltage-dependent calcium channels as a prerequisite of neuronal excitation, modulation of intracellular signaling pathways and vesicular release of neurotransmitters (glutamate) and discharge of neurohormones (GnRH, galanin). Some members of the potassium channel proteins, belonging mainly to the voltage-gated and inwardly rectifying subfamily, also established a protein cluster with heavy links with the sodium and calcium protein groups. The functional clarification of this proestrus-evoked response awaits further studies.

Proestrus also evoked down regulation of some channel proteins that formed one single cluster. It was composed by mixed calcium and potassium channel protein subunits. In case of the calcium channel, the response was manifested in 3 subunits of the voltage-dependent calcium channels. The downregulated potassium channel proteins belonged to the K-type subfamily and the voltage-gated potassium channel class. The downregulation of these potassium channel components may support the increased activity of GnRH neurons at late proestrus.

## Conclusion

In this study, we elucidated the expression profile of ion channel genes in GnRH neurons of regularly cycling mice processed before the onset of the GnRH surge. The dataset allows insight into the putative remodeling of the different channels. Although, E2 is a key hormone in the positive gonadal steroid feedback acting on GnRH neurons and their afferent neurons, other ovary-born hormones (inhibin, anti-Mullerian hormone and others) also have regulatory effects (Adams et al., 2018). Therefore, we have put the emphasis on the impact of proestrus, instead of E2. Regarding the molecular events, we explored significant differences in expression of genes encoding sodium, potassium, and chloride and calcium ion channel-forming proteins in GnRH neurons obtained from pro- and metestrous mice, respectively. The differential expression of ion channel-coding genes in proestrus elucidates the subtypes of ion channels that contribute to the altered electrophysiology and function of GnRH neurons prior to the GnRH surge.

## Author Contributions

CV designed and performed the experiments and analyzed the data. NS carried out the bioinformatical analysis of the microarray data. IF contributed to discussion of electrophysiological significance of findings. ZL designed and supervised the project and wrote the manuscript.

## Conflict of Interest Statement

The authors declare that the research was conducted in the absence of any commercial or financial relationships that could be construed as a potential conflict of interest.

## Footnotes

1 http://www.genome.jp/kegg/

2 https://string-db.org
